# Many-Objective Quantum-Inspired Particle Swarm Optimization Algorithm for Placement of Virtual Machines in Smart Computing Cloud

**DOI:** 10.3390/e24010058

**Published:** 2021-12-28

**Authors:** Jerzy Balicki

**Affiliations:** Faculty of Mathematics and Computer Science, Warsaw University of Technology, 00-662 Warsaw, Poland; jerzy.balicki@pw.edu.pl

**Keywords:** particle swarm optimization, quantum gates, virtual machines, computing cloud, many-objective optimization

## Abstract

Particle swarm optimization algorithm (PSO) is an effective metaheuristic that can determine Pareto-optimal solutions. We propose an extended PSO by introducing quantum gates in order to ensure the diversity of particle populations that are looking for efficient alternatives. The quality of solutions was verified in the issue of assignment of resources in the computing cloud to improve the live migration of virtual machines. We consider the multi-criteria optimization problem of deep learning-based models embedded into virtual machines. Computing clouds with deep learning agents can support several areas of education, smart city or economy. Because deep learning agents require lots of computer resources, seven criteria are studied such as electric power of hosts, reliability of cloud, CPU workload of the bottleneck host, communication capacity of the critical node, a free RAM capacity of the most loaded memory, a free disc memory capacity of the most busy storage, and overall computer costs. Quantum gates modify an accepted position for the current location of a particle. To verify the above concept, various simulations have been carried out on the laboratory cloud based on the OpenStack platform. Numerical experiments have confirmed that multi-objective quantum-inspired particle swarm optimization algorithm provides better solutions than the other metaheuristics.

## 1. Introduction

An approach based on many-objective decision-making can be developed for smart computer infrastructures in some crucial domains such as education, health care, public transport and urban planning. If the number of criteria is greater than 3, we consider the many-criteria optimization problem as a special case of multi-criteria optimization problem. However, more importantly, as the number of criteria increases, there is an explosion in the number of Pareto-optimal solutions. We show an example where the number of effective evaluations is five for two criteria and then two hundred for four criteria. So, how much will it be for the seven criteria and more? In this paper, we explain this phenomenon of a sudden explosion of the number of Pareto-optimal solutions. Consequently, much more memory should be allocated to the Pareto solution archive and a much larger population size should be assumed in evolutionary algorithms, particle swarm optimization (PSO) or ant colony optimization (ACO).

Because several criteria characterize complex systems such as computing clouds, the placement of virtual machines in smart computing clouds is formulated as the many-optimization problem. In the model of the multi-task training, the selected deep learning models (DLs) can be retrained at the central hosts cyclically, and then their copies migrate as virtual machines (VMs) to an edge of the computing cloud. Besides this, a host failure forces the virtual machines to be moved immediately from that computer to the most appropriate servers. This smart computing cloud should be supported by using teleportation of virtual machines via the Internet of Things (IoT) to optimize several criteria such as energy consumption, the workload of the bottleneck computer, cost of hardware, reliability of the cloud and others. Some criteria can be constrained because of the dramatic increase in wireless devices that influence several additional bounds [1].

Recent advances in cloud computing, deep learning and Big Data have introduced data-driven solutions into platforms based on OpenStack, which enables the construction of private computing clouds with their own intelligent services, for example, for education at universities or for the smart city. On the long side, commercial public clouds such as Microsoft Azure and Amazon Elastic Compute Cloud immediately deliver advanced services based on artificial intelligence and quantum computing. In both cases, efficient placement of VMs and management of computer resources is one of the particularly important issues [2]. An adequate resource assignment is a crucial challenge for optimization of teleportation of virtual machines by a cloud hypervisor that allocates computer resources to virtual machines with requirements due to central processing units (CPUs), graphical processing units (GPUs), random-access memory (RAM) and disk storage. Live migration is very effective because it involves transferring VMs without shutting down client systems for clouds and without doing a lot of administrative work [3]. 

Teleportation of a virtual machine can change the workload of the bottleneck computer or the workload of the bottleneck transmission node. Besides this, the energy consumption by the set of cloud hosts depends on the virtual machine assignment. Some VMs can be moved to less energy-consuming hosts, and the others can be allocated to database servers to accelerate operations on their data. Therefore, a problem of cloud resource assignment should be formulated as a multi-objective optimization issue with selected criteria. We present the new many-objective problem of virtual machine placement with seven criteria such as: electric power of hosts, reliability of cloud, CPU workload of the bottleneck host, communication capacity of the critical node, a free RAM capacity of the most loaded memory, a free disc memory capacity of the most busy storage, and overall computer costs. This is a significant extension of the current formulation of this issue with four criteria [4]. 

Metaheuristics such as genetic algorithms, differential evolution, harmony search, bee colony optimization, particle swarm optimization and ant colony algorithms can be used for solving multi-criteria optimization problems to find the representation of Pareto-optimal solutions [5]. Based on many numerical experiments, we propose Many-objective Quantum-inspired Particle Swarm Optimization Algorithm (MQPSO) with additional improvements based on quantum gates. Not only do high-quality solutions justify this approach, but also that decision-making in computing clouds requires quantum-inspired intelligent software to handle some dynamic situations. Furthermore, PSOs have been applied to support several key tasks in computer clouds.

A goal of this paper is to study MQPSO for optimization of VMs placement regarding selected seven criteria subject to the computer resource constraints. One of the main contributions is the formulation of the many-objective optimization problem with seven crucial criteria for the management of virtual machines, and the MQPSO algorithm that can be used to determine the Pareto-optimal solutions for the other many-objective optimization problems, too.

To order many important issues, the rest of this paper is organized, as follows. Related work is described in Section 2. Then, a teleportation model of virtual machines is characterized in Section 3. Next, Section 4 presents many-objective optimization problem formulation, and then Section 5 describes MQPSO with quantum gates for finding Pareto-optimal solutions. Finally, some numerical experiments are presented in Section 6 just before Section 7 with conclusions.

## 2. Related Work

We consider studies on the placement of virtual machines in the computing cloud, and then we analyze quantum-inspired PSOs [6]. An overview of virtual machine placement schemes in cloud computing is presented in [7]. An energy-efficient algorithm for live migration of virtual machines may reduce wastage of power by initiating a sleep mode of idle hosts [8]. To avoid overloaded servers, the cloud workload optimizer analyzes load on physical machines and determines an optimal placement due to energy consumption by an ant colony optimization algorithm. A local migration agent based on an optimal solution selects appropriate physical servers for VMs. Finally, the migration orchestrator moves the VMs to the hosts, and an energy manager initiates a sleep mode for idle hosts to save energy. In addition, efficient managing of resources is recommended for green (or energy-aware) cloud with parallel machine learning [9]. 

It is a very small (almost zero) downtime in the order of milliseconds within live migration of virtual machines [1]. This live migration is carried out without disrupting any active network connections, even when the VM is moved to the target host because an original VM is running. There are other benefits associated with migrating VMs. For instance, the placement of VMs can be a solution for the minimization of the processor usage of hosts or for the minimization of their Input/Output usage subject to keeping virtual machines zero downtime [10]. To increase the throughput of the system, VMs are supposed to be distributed to each server in proportion to their computing Input/Output capacity. Migrations of virtual machines can be conducted by the management of services of software platforms such as Red Hat Cluster Suite to create load balancing cluster services [10].

We recommend OpenStack as the suited cloud software for supporting the live migration of VMs. For instance, it was demonstrated by using high network interfaces with transmission capacity10 Gb/s [11]. Our experimental cloud WUT-GUT confirms the adequate possibilities of OpenStack due to the teleportation of VMs.

An influence of both the virtual machine size for transmission and bandwidth of the network on required teleportation time is very crucial [9]. We can save energy by well-organized management of these resources for cloud data centers. Besides this, an adequate placing VM in datacenters may diminish the number of VM migrations that is another optimization criterion [12]. If the smart VMs are going to migrate, some of them may be unable to obtain the destination hosts due to the limited resources. To avoid the above situations, Wang et al. formulated another NP-hard optimization problem, which both maximizes the revenue of successfully placing the VMs and minimizes the total number of migrations subject to the resource constraint of hosts. Regarding complexity proof, the above problem is equivalent to finding the shortest path problem [9].

Deep reinforcement learning was developed for multi-objective placement of virtual machines in cloud datacenters [13]. Because high packing factors could lead to performance and security issues, for example, virtual machines can compete for hardware resources or collude to leak data, it was introduced a multi-objective approach to find optimal placement strategies considering different goals, such as the impact of hardware outages, the power required by the datacenter, and the performance perceived by users. Placement strategies are found by using a deep reinforcement learning framework to select the best placement heuristic. A proposed algorithm outperforms bin packing heuristics [13].

Task scheduling and allocation of cloud resources impact the performance of applications [14]. Customer satisfaction, resource utilization and better performance are crucial for service providers. A multi-cloud environment may significantly reduce the cost, makespan, delay, waiting time and response time to avoid customer dissatisfaction and to improve the quality of services. A multi-swarm optimization model can be used for multi-cloud scheduling to improve the quality of services [14].

Green strategies for networking and computing inside data centers, such as server consolidation or energy-aware routing, should not negatively impact the quality and service level agreements expected from network operators [15]. Therefore, robust strategies can place virtual network functions to save energy savings and to increase the protection level against resource demand uncertainty. The proposed model explicitly provides for robustness to unknown or imprecisely formulated resource demand variations, powers down unused routers, switch ports and servers and calculates the energy optimal virtual network functions placement and network embedding also considering latency constraints on the service chains [15]. A fast robust optimization-based heuristic for the deployment of green virtual network functions was studied in [16].

Because a service function chain specifies a sequence of virtual network functions for user traffic to realize a network service, the problem of orchestration this chain is very crucial [17]. It can be formulated the deadline-aware co-located and geo-distributed orchestration with demand uncertainty as optimization issues with the consideration of end-to-end delay in service chains by modeling queueing and processing delays. The proposed algorithm improved the performance in terms of the ability to cope with demand fluctuations, scalability and relative performance against other recent algorithms [17].

Another aspect of virtualization is the nonlinear optimization problem of mapping virtual links to physical network paths under the condition that bandwidth demands of virtual links are uncertain [18]. To realize virtual links with predictable performance, mapping is required to guarantee a bound on the congestion probability of the physical paths that embed the virtual links. 

The second part of the work is about some new approaches to PSO algorithms. Although quantum-inspired PSO algorithms have not been used to optimize the migration of virtual machines in the computing cloud so far, it is worth discussing them due to the PSO algorithm with quantum gates proposed in the article. The quantum-behavior particle swarm optimization (QPSO) algorithm may control placement problems in software-defined networking to make computer networks agile and flexible [19]. To meet the requirements of users and conquer the physical limitation of networks, it is necessary to design an efficient controller placement mechanism, which is defined as an optimization problem to determine the proper positions and number of its controllers. A particle is a vector for placing controllers at each switch. Besides this, the algorithm is characterized by quantum behavior with no quantum gates. QPSO algorithm demonstrates power fast convergence rate but limits in global search ability. Simulation results show that QPSO achieves better performance in several instances of multi-controller placement problems [19]. 

An enhanced quantum behaved particle swarm optimization (e-QPSO) algorithm improves the exploration and the exploitation properties of the original QPSO for function optimization. It is based on the adaptive balance among the personal best and the global best positions using the parameter alpha. Besides this, it keeps the balance between diversification and intensification using the parameter gamma. In addition, a percentage of the worst-performing population is re-initialized to prevent the stack of the particle at the local optima. The results of e-QPSO outperform twelve QPSO variants, two adaptive variants of PSO, as well as nine well-known evolutionary algorithms due to 59 benchmark instances [6].

A self-organizing quantum-inspired particle swarm optimization algorithm (MMO_SO_QPSO) is another version of PSO for solving multimodal multi-objective optimization problems (MMOPs) [20]. It should find all equivalent Pareto optimal solutions and maintain a balance between the convergence and diversity of solutions in both decision space and objective space. In the algorithm, a self-organizing map is used to find the best neighbor leader of particles, and then a special zone searching method is adopted to update the position of particles and locate equivalent Pareto optimal solutions in decision space. Quantum behaviors of particles are not described as position vectors and velocity vectors, but they are replaced by the wave function. To maintain diversity and convergence of Pareto optimal solutions, a special archive mechanism based on the maximum-minimum distance among solutions is developed. Some outstanding Pareto optimal solutions are maintained in another archive. In addition, a performance indicator estimates the similarity between obtained Pareto optimal solutions and true efficient alternatives. Experimental results demonstrate the superior performance of MMO_SO_QPSO for solving MMOPs [20]. 

It is worth noting that the MMO_SO_QPSO algorithm is one of the most advanced PSO algorithms inspired by quantum particle behavior. For this reason, it is the foundation on which our algorithm MQPSO is based, which additionally takes into account quantum gates and the many criteria selection procedure. 

Besides this, there are several interesting works that have dealt with hybrid metaheuristics, which could provide an effective introduction to advanced metaheuristics in general [21], in combination with exact approaches applied in network design [22] and improved particle swarm optimization [23].

## 3. Live Migration of Intelligent Virtual Machines

We can identify the following system elements that are relevant for the modeling. Let *V* be the number of virtual machines that are trained in the cloud on *I* physical machines. Moreover, let virtual machines be denoted as *α*_1_, …, *α_v_*, …, *α_V_*. Each *α_v_* can work at the host assigned to the safety node such as *N*_1_, …, *N_i_*, …, *N_I_* in the cloud. Let *β*_1_, …, *β_j_*, …, *β_J_* be the possible types of physical machines providing a set of resources to VMs. Exactly one physical machine *β_j_* can be assigned to each node *N_i_*.

Virtual machine migration involves changing the host on the source node to the host on the target node, which is a key decision in the optimization problem under consideration. Destination nodes of the VMs after migrations can be modeled by $X^{\alpha }={\left[x_{vi}^{\alpha }\right]}_{V\times I}$, where [4]:

(1) $$x_{vi}^{\alpha }=\{\begin{array}{c} 1\;\mathrm{if}\;\alpha_{v}\;\mathrm{is}\;\mathrm{assigned}\;\mathrm{to}\;\mathrm{the}\;\mathrm{node}\;N_{i},\\ 0\;\mathrm{in}\;\mathrm{the}\;\mathrm{other}\;\mathrm{case},\; \end{array}\;v=\bar{1,V},\;i=\bar{1,I}$$

To find adequate amounts of resources, we consider the assignment of hosts that are the other important decisions supporting the placement of VMs. Capacities of resources in the cloud can be adapted to the needs of virtual machines by designating an appropriate matrix $X^{\beta }={\left[x_{ij}^{\beta }\right]}_{I\times J}$, where:

(2) $$x_{ij}^{\beta }=\{\begin{array}{c} 1\;\mathrm{if}\;\beta_{j}\;\mathrm{is}\;\mathrm{assigned}\;\mathrm{to}\;\mathrm{the}\;\mathrm{node}\;N_{i},\\ 0\;\mathrm{in}\;\mathrm{the}\;\mathrm{other}\;\mathrm{case},\; \end{array}\;i=\bar{1,I},\;j=\bar{1,J}$$

Resources provided by physical machines are characterized by six vectors such as: the vector of electric power consumption $\varepsilon =\left[\varepsilon_{1},\ldots ,\varepsilon_{j},\ldots ,\varepsilon_{J}\right]$ (watt), the vector of RAM memory capacities $ram=\left[ram_{1},\ldots ,ram_{j},\ldots ,ram_{J}\right]$ (GB), the vector $\nu =\left[\nu_{1},\ldots ,\nu_{j},\ldots ,\nu_{J}\right]$ of the numbers of the preferred computers and the vector of disk storage capacities $hdd=\left[hdd_{1},\ldots ,hdd_{j},\ldots ,hdd_{J}\right]$ (TB). In addition, the vector $\xi =\left[\xi_{1},\ldots ,\xi_{j},\ldots ,\xi_{J}\right]$ ($) characterizes costs of using the selected physical machines. Let the computer $\beta_{j}$ be failed independently due to an exponential distribution with rate $\theta_{j}$. The sixth vector is the vector of the reliability rates $\theta =\left[\theta_{1},\ldots ,\theta_{j},\ldots ,\theta_{J}\right]$.

Moreover, four characteristics are related to virtual machines. Let $T={\left[t_{vj}\right]}_{V\times J}$ be the matrix of cumulative times of VMs runs on different types of computers (s) and also $\tau ={\left[\tau_{vuik}\right]}_{V\times V\times I\times I}$—the matrix of total communication time between agent pairs located at different nodes (s). In addition, the memory requirements of VMs are described by the vector $r=\left[r_{1},\ldots ,r_{v},\ldots ,r_{V}\right]$ of reserved RAM (GB) and the vector $h=\left[h_{1},\ldots ,h_{v},\ldots ,h_{V}\right]$ of required disk storage (TB). 

The binary matrix double $x=\left(X^{\alpha },\;X^{\beta }\right)$ considers decision variables that can minimize four criteria and maximize three criteria characterizing computing cloud. We maximize three criteria. Let $\kappa_{min}^{RAM}$ be the free capacity of the RAM in the bottleneck computer due to RAM (GB) and $\kappa_{min}^{disc}$—the free capacity of the disc storage in the bottleneck computer due to disk storage (TB) [4]. Let *R* denote the reliability of the cloud that should be maximized, too. Moreover, we minimize *E*—electric power of the cloud (watt). Let ${\tilde{Z}}_{max}$ be communication capacity of the bottleneck node (s). Moreover, *Ξ*—cost of hosts (money unit, i.e., $) is the criterion for minimization. Let ${\hat{Z}}_{max}$ be the processing workload of the bottleneck CPU (s) that is the fourth criterion for maximization.

Live migration of virtual machines may change the values of the above criteria. Therefore, we should find the set of Pareto-optimal solutions. On the other hand, the decision-maker can choose a compromise solution from this set. 

Intelligent virtual machines contain some pre-trained domain models based mainly on convolutional neural network (CNN) and long short term memory (LSTM) ANNs [24,25]. Several virtual machines can run on a host, but they can migrate from the source computer to the destination regarding the Pareto-optimal solution obtained by the MQPSO. For instance, an accuracy of 99% can be achieved within approximately one minute for training the CNN on the virtual machine Linux Fedora Server (the CPU Intel Core i7, 27 GHz, RAM 16 GB) due to the German Traffic Sign Detection Benchmark Dataset. The CNN identifies the traffic signs represented by the matrix with 28 × 28 selected features. The dataset is divided into 570 training signs and 330 test items [24]. In this case, live migration of this virtual machine is unlikely to be recommended, unless there is a host failure [26]. Retraining is very fast, and then a trained model can be sent to nodes on the Internet of Things [27].

However, a virtual machine needs additional resources in the following situation, which is very common in intelligent clouds [28]. Much more time is required to learn the Long Short Term Memory artificial neural network for video classification. The LSTM can detect some dangerous situations from the web camera monitoring city areas using the Cityscapes Dataset with 25,000 stereo videos about the street scenes from 50 cities [29]. After 70 h of the CPU elapsed time, LSTM based model achieved an average accuracy of 54.7% that is rather unacceptable. The LSTM was implemented at the Matlab R2021b environment. Therefore, the supervised learning process should be accelerated by using GPUs, and the virtual machine with LSTM is supposed to be moved to the other server.

Live migration of the virtual machine to the more powerful workstation with the GPUs can be carried out by using protocols HTTPS and WebSocket. Besides this, micro-services exchange data with format JSON, which were verified in the experimental computing cloud GUT-WUT (Gdańsk University of Technology—Warsaw University of Technology). This cloud is based on the OpenStack software platform that maintains possibilities of VM teleportation [4]. 

Another deep learning model that uses the virtual machine may be the Human Motion DataBase (HMDB) with 6849 clips divided into 51 action categories, each containing a minimum of 101 clips [30]. For instance, the trained LSTM can detect undesirable situations in the city, such as smoking and drinking in the forbidden areas or pedestrians falling on the floor. The quick and correct classifications allow counteracting many extreme situations on city streets [31]. Teleportation of the virtual machine with the LSTM trained on the HMDB can provide enough amount of cloud resources to train this virtual machine with high accuracy. 

## 4. Many-Objective Optimization Problem

There are the upper constraints that have to be respected to guarantee project requirements such as the maximum computer load ${\hat{Z}}_{sup}$ or an upper limit of node transmission ${\tilde{Z}}_{sup}$. To save energy, let $\xi_{max}$ be limit of electric power for the cloud. A budget constraint for the project can be denoted as $\varepsilon_{max}$. Furthermore, there are minimal requirements for three maximizing metrics. *R_min_* is the minimum reliability for the hosts used by virtual machines. $\kappa_{inf}^{disc}$ represents the free capacity of the disc storage in the bottleneck computer due to disk storage (TB). $\kappa_{inf}^{RAM}$ is the free capacity of the RAM in the bottleneck computer due to RAM (GB). We can establish constraints, as below:

(3) $$Ε\left(x\right)\leq \varepsilon_{max}$$

(4) $$\kappa_{min}^{RAM}\left(x\right)\geq \kappa_{min}^{RAM}$$

(5) $$\kappa_{min}^{disc}\left(x\right)\geq \kappa_{min}^{disc}$$

(6) $$R\left(x\right)\geq R_{min}$$

(7) $${\hat{Z}}_{max}\left(x\right)\leq {\hat{Z}}_{sup}$$

(8) $${\tilde{Z}}_{max}\left(x\right)\leq {\tilde{Z}}_{sup}\;$$

(9) $$\Xi \left(x\right)\leq \xi_{max}$$

If we rebuild the cloud, some hosts can be removed from it, but the others can be left to cooperate with the new servers. Therefore, from the current set of computers $C^{now}$ we can determine the set of preferred computer types $B^{now}$. Let $J^{now}$ be the set of indexes of the preferred computer types for the current infrastructure. Moreover, we are going to buy or rent the new hosts. Let $J^{new}$ be the set of indexes of the new computer types. Let $J=J^{now}\cup J^{new}$, where $J={\left\{1,\ldots ,j,\ldots ,J\right\}}^{}$. If we consider the new servers, we can buy 0, 1, 2 or more hosts of the same kind. However, we are supposed to respect the assumed computers number *ν_j_* of the *j*th type from $J^{now}.$

In consequence, the following constraint is introduced:

(10) $$\sum_{i=1}^{I}x_{ij}^{\beta }=\nu_{j},j\in J^{now}$$

The reliability *R* is defined, as below [4]:

(11) $$R\left(x\right)=\prod_{v=1}^{V}\prod_{i=1}^{I}\prod_{j=1}^{J}e^{-\theta_{j}t_{vj}x_{vi}^{\alpha }x_{ij}^{\beta }}$$

To calculate the workload of the bottleneck computer, we introduce two formulas. If computer *β_j_* is located in the cloud node no. *i*, the decision variable $x_{ij}^{\beta }$ is equal to 1. If virtual machine no. *v* is included in this cluster, the decision variable is equal to 1, too. The term $t_{vj}x_{vi}^{\alpha }x_{ij}^{\beta }$ is the cumulative time of the *v*th virtual machine run on computer *β_j_* (s). Now, we can calculate the workload of the bottleneck computer, as follows [4]:

(12) $${\hat{Z}}_{max}\left(x\right)=\underset{i=\bar{1,I}}{\max}\left\{\sum_{j=1}^{J}\sum_{v=1}^{V}t_{vj}x_{vi}^{\alpha }x_{ij}^{\beta }\right\}\;$$

Similarly, we can determine transmission workload of the bottleneck node by the following formula:

(13) $${\tilde{Z}}_{max}\left(x\right)=\underset{i=\bar{1,I}}{\max}\left\{\sum_{v=1}^{V}\sum_{\begin{array}{c} u=1\\ u\neq v \end{array}}^{V}\sum_{i=1}^{I}\sum_{\begin{array}{c} k=1\\ i\neq k \end{array}}^{I}\tau_{vuik}x_{vi}^{\alpha }x_{uk}^{\beta }\right\}$$

If computer *β_j_* is located in the cloud node no. *i*, electric consumption per time unit in this node is equal to $\varepsilon_{j}x_{ij}^{\beta }$, and all nodes in this cloud require electric power, as below:

(14) $$E\left(x\right)=\sum_{i=1}^{I}\sum_{j=1}^{J}\varepsilon_{j}x_{ij}^{\beta }\;$$

Also, we can calculate the total cost of computers, as follows:

(15) $$\Xi \left(x\right)=\sum_{i=1}^{I}\sum_{j=1}^{J}\xi_{j}x_{ij}^{\beta }\;$$

Because the cluster of agents requires the RAM capacity $\sum_{v=1}^{V}r_{v}x_{vi}^{\alpha }$ in node no. *i*, the minimal capacity of RAM memory for a bottleneck host can be calculated, as follows:

(16) $$\kappa_{min}^{RAM}\left(x\right)=\underset{i=\bar{1,I}}{\min}\left\{\sum_{j=1}^{J}ram_{j}x_{ij}^{\beta }-\sum_{v=1}^{V}r_{v}x_{vi}^{\alpha }\right\}$$

Likewise, we can identify the bottleneck computer regarding free disc storage in the cloud:

(17) $$\kappa_{min}^{disc}\left(x\right)=\underset{i=\bar{1,I}}{\min}\left\{\sum_{j=1}^{J}hdd_{j}x_{ij}^{\beta }-\sum_{v=1}^{V}h_{v}x_{vi}^{\alpha }\right\}$$

Based on the model, we can formulate the many-criteria optimization problem, as follows.

**Given**: 

numbers *V*, *I*, *J*, $\varepsilon_{max},\;$*R_min_*, $\kappa_{inf}^{disc},$ $\kappa_{inf}^{RAM},\;{\hat{Z}}_{sup},\;{\tilde{Z}}_{sup},\;\xi_{max},$

vectors ε, ram, hdd, ξ, ν,θ, r, h

matrices T, τ

**Find** the representation of Pareto-optimal solutions $X^{Pareto}$ for an ordered tuple:

(18) $$\left(X,\;F,\;{⥼}_{\leq }\right)$$

where:

(1) X—the set of admissible solutions *x* that satisfy requirements (3)–(10) and the formal constraints, as below:

(19) $$\sum_{i=1}^{I}x_{vi}^{\alpha }=1,\;v=\bar{1,V,}$$

(20) $$\sum_{j=1}^{J}x_{ij}^{\beta }=1,\;i=\bar{1,I},$$

(2) F—the vector of seven minimized partial criteria

(21) $$F:X\to R^{7}\;$$

(22) $$F\left(x\right)=[\;{\hat{Z}}_{max}\left(x\right),\;{\tilde{Z}}_{max}\left(x\right),\;Ε\left(x\right),\Xi \left(x\right),-\kappa_{min}^{RAM}\left(x\right),-\kappa_{min}^{disc}\left(x\right),-R\left(x\right)]\;$$

R—a set of real numbers

(3) ${⥼}_{\leq }$—the domination relation in ***R***^7^

(23) $${⥼}_{\leq }=\left\{\left(a,\;b\right)\in Y\times Y|a_{n}\leq b_{n},\;n=\bar{1,7}\right\},\;Y=F\left(X\right)$$

The number of the admissible solutions $x=\left(X^{\alpha },\;X^{\beta }\right)$ is no greater than 2*^(V+J)I^*. If the binary encoding of solutions is substituted by the integer encoding, the upper limit of admissible solutions is *V ^I^ I ^J^*. In the integer encoding, we introduce the modified decision variables: $X_{v}^{\alpha }=i\;\mathrm{if}\;x_{vi}^{\alpha }=1,$ $X_{i}^{\beta }=j\;\mathrm{if}\;x_{ij}^{\beta }=1$. In such a way, the formal constraints are satisfied. Let *n* ≈ *V* ≈ *I* ≈ *J*. The upper limit of admissible solutions increases in a non-polynomial way due to *O*(*n^n^*). We can prove the following Lemma.

**Lemma** **1.** *If number of nodes I ≥ 4 or I ≥ 2 and memory resources are limited, then the many-criteria combinatorial optimization problem (18)–(23) is NP-hard.*

**Proof.**  We will prove that the problem (18)–(23) is polynomially reducible to any known NP-hard problem. Let some assumptions be made to transform the formulated dilemma (18)–(23) into the other NP-hard issue. If *J* = 1, then we consider the placement of *V* virtual machines on *I* computers. Moreover, let resource constraints are released and one objective function ${\hat{Z}}_{max}$ is considered, only. Then, this case of the problem (18)–(23) is equivalent to a task assignment problem without memory limits [32]. If *I* ≥ 4, it was proved that task assignment dilemma without constraints is NP-hard for minimization of the total cost [32]. On the other hand, if *I* ≥ 2 and memory resources are limited, then minimization of the total cost for task assignment is NP-hard, too [32]. Each hierarchical solution related to minimization ${\hat{Z}}_{max}$ is Pareto-optimal solution [4]. Because the problem (18)–(23) with one criterion is NP-hard, the extended issue with seven criteria is NP-hard, which ends the proof. □

Note that the problem of finding a minimum feasible assignment in some cases is equivalent to a knapsack problem, and hence is an NP-hard problem. Consider the *I* node graph, in which every node is of degree 2 and the source and the sink both have degrees (*I*-2). The weight *w_i_* of the node *N_i_* corresponds to the weight of the *i*th item in the knapsack problem. A feasible cut of the task assignment graph corresponds to a subset of items whose weights do not exceed the knapsack constraint weight *w*. A minimum feasible cut corresponds to a knapsack packing of maximum value [32].

Solving problem (18)–(23) by an enumerative algorithm is ineffective for the large search space with *V^I^ I^J^* elements. Let us consider the instance of the problem (18)–(23) with 855 decision variables. In the experimental instance called Benchmark855 (https://www.researchgate.net/publication/341480343_Benchmark_855, accessed: 19 November 2021), an algorithm determines a set of Pareto-optimal solutions for 45 virtual machines, 15 communication nodes and 12 types of servers. A search space contains 8.2 × 10^38^ elements and 2 × 10^7^ solutions are evaluated during 3 min by an enumerative algorithm implemented in Java on Dell E5640 dual-processor machine under Linux CentOS. It confirms that there are no practical chances to find any Pareto-solution by a systematic enumerative way. Besides this, 2 × 10^7^ independent probabilistic trials are less likely to ensure a high-quality alternative. 

There are exact solvers like the multi-criteria branch and bound method [33] or the ε-constraint method [34], but they usually produce poor quality solutions for the limited time of calculations. Metaheuristics find much better results than exact methods for many instances of different NP-hard multi-criteria optimization problems [21]. 

## 5. Many-Objective Particle Swarm Optimization with Quantum Gates MQPSO

We simulated teleportation of virtual machines at the cloud GUT-WUT based on OpenStack that uses hosts from two universities [4]. Algorithm 1 shows pseudocode visualizing how the various steps of the general algorithm many-objective particle swarm optimization with quantum gates (MQPSO) are adapted to the specific features of the VMs placement problem (18)–(23). The algorithm is based on Hadamard gates and rotation gates. Hadamard gate converts a qubit of a quantum register Q into a superposition of two basis states ∣0⟩ and ∣1⟩, as follows [35]:
**Algorithm 1** Multi-objective Quantum-inspired PSO1:Set input data, *A*(t) := Ø; *t* := 02:Initialize quantum register *Q*(*t*) with *M* qubits by the set of Hadamard gates3:**while** (**not** termination condition) **do**4: create *P*(*t*) by observing the state of *Q*(*t*)5: determine new positions and velocities of particles followed by create *B*(*t*)6: find Fonseca-Fleming ranks for an extended archive *C*(*t*) = *A*(*t*)∪*B*(*t*)∪*P*(*t*)7: calculate crowding distances, fitness and then sort particles in *C*(*t*)8: form *A*(*t*) of Pareto-optimal solutions from the sorted set *C*(*t*)9: a tournament selection of an angle rotation matrix $M_{\theta }$ based on rating $R\left(M_{\theta }\right)$10: mutate the selected matrix $M_{\theta }$ with the rate *p_m_*11: modify *Q*(*t*) using the rotation gates12: *t* := *t* + 113:**end while**

(24) $$|0\rangle =\{\begin{array}{c} \frac{\left|0\rangle +\right|1\rangle }{\sqrt{2}}\;\mathrm{for}\;\mathrm{the}\;\mathrm{basis}\;\mathrm{state}\;|0\rangle \\ \frac{\left|0\rangle -\right|1\rangle }{\sqrt{2}}\;\mathrm{for}\;\mathrm{the}\;\mathrm{basis}\;\mathrm{state}\;|1\rangle \end{array}\;$$

The Hadamard gate is a single-qubit operation based on the 90° rotation around the *y*-axis, and then a 180° rotation around the *x*-axis. If we use Dirac notation for the description of the qubit state $Q_{m}=\alpha_{m}|0\rangle ⊕\beta_{m}|1\rangle$, the qubit can be represented by the matrix, as follows [6]:

(25) $$Q=\left[\begin{array}{ccccc} \left|\alpha_{1}\right| & \ldots & \left|\alpha_{m}\right| & \ldots & \left|\alpha_{M}\right|\\ \left|\beta_{1}\right| & \ldots & \left|\beta_{m}\right| & \ldots & \left|\beta_{M}\right| \end{array}\right]$$

The procedure of random selection of decision values is involved with a chromosome matrix. If the decision variable *x_m_* is characterized by a pair of complex numbers (*α_m_*, *β_m_*), it is equal to 0 with the probability ${\left|\alpha_{m}\right|}^{2}$ and to 1 with ${\left|\beta_{m}\right|}^{2}$. Alternatively, the state of the *m*th qubit can be represented as the point on the 3D Bloch sphere, as follows [35]:

(26) $$|Q_{m}\rangle =\cos\frac{\theta_{m}}{2}\;|0\rangle +e^{i\phi_{m}}\sin\frac{\theta_{m}}{2}\;\;|1\rangle ,\;m=\bar{1,M},$$

where $0\;\leq \;\theta_{m}\leq \pi$ and $0\leq \;\phi_{m}\leq 2\pi$.

In the 3D Bloch sphere, the Hadamard gate can be implemented by several rotations to achieve the desired point determined by a pair of angles ($\theta_{m},\;\phi_{m}$), that is, equal superposition of the two basis states. Two angles $\theta_{m}$ and $\phi_{m}$ determines the localization of qubit on the Bloch sphere. The North Pole represents the state |0⟩, the South Pole represents the state |1⟩ and the points on the equator represent all possible states in which 0 and 1 are the same. Thus, in this version of the quantum-inspired genetic algorithm, there are *M* Bloch spheres with the quantum gene states.

Therefore, the Hadamard gate can be modeled, as the matrix operation, as below:

(27) $$H=\frac{1}{\sqrt{2}}\left[\begin{array}{cc} 1 & 1\\ 1 & -1 \end{array}\right]$$

The Hadamard gate can be implemented by the Pauli gates. The Pauli-X gate (*PX*) is a single-qubit rotation through π radians around the *x*-axis. On the other hand, the Pauli-Y gate (*PY*) is a single-qubit rotation through π radians around the *y*-axis. From (27), we get the following:

(28) $$H=PX\cdot PY^{\frac{1}{2}}=PY^{-\frac{1}{2}}\cdot PX,$$

where:

$PX=\left[\begin{array}{cc} 0 & 1\\ 1 & 0 \end{array}\right]$,
$PY=\left[\begin{array}{cc} 0 & -i\\ i & 0 \end{array}\right]$,
*i*—the imaginary unit of a complex number.

For the Pauli-Z gate (*PZ*) that is a single-qubit rotation through π radians around the *z*-axis, we have, as below:

(29) $$H=ZX\cdot PY^{\frac{1}{2}}=PY^{-\frac{1}{2}}\cdot ZX,$$

where $ZX=\left[\begin{array}{cc} 1 & 0\\ 0 & -1 \end{array}\right]$.

The initial step of MQPSO (Algorithm 1, step 1) is to enter data such as *V*, *I*, *J*, $\varepsilon_{max},$ *R_min_*, $\kappa_{inf}^{disc},$ $\kappa_{inf}^{RAM},$ ${\hat{Z}}_{sup},\;{\tilde{Z}}_{sup},\;\xi_{max},\;\varepsilon ,\;ram,\;hdd,\;\xi ,\;\nu ,\theta ,\;r,\;h,\;T,\;\tau .$ Then, the initial value of the main loop is set to 0 (t := 0). 

**Figure 1:**
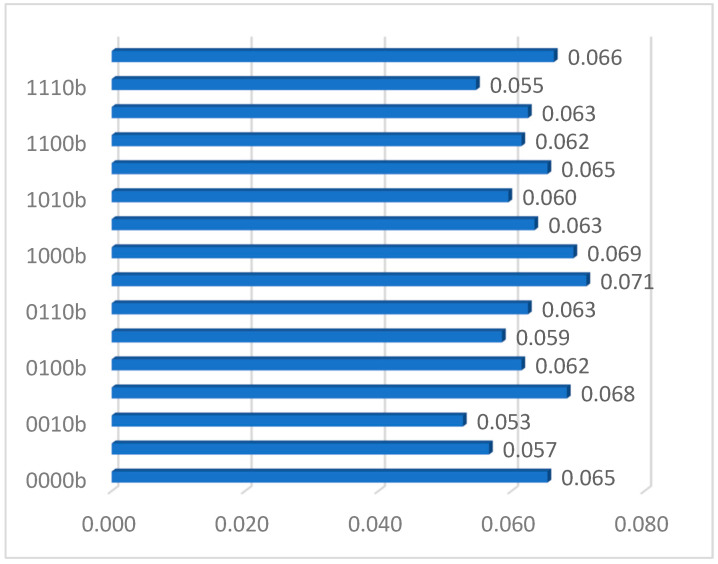
Probability distribution of the virtual machine placement after quantum register measurement.

The quantum register *Q*(*t*) consists of *M* qubits (Algorithm 1, step 2). The *M* Hadamard gates are used, concurrently. We consider (*V* + *I*) blocks of qubits representing decision variables $X_{v}^{\alpha },\;v=\bar{1,V},\;\mathrm{and}\;X_{j}^{\beta },\;j=\bar{1,J}$. If there are *λ* quantum bits for encoding decisions $X_{V}^{\alpha }$ and *μ* quantum bits for encoding $X_{j}^{\beta }$, there are *M* = λV+μI quantum bits for the register *Q*. To minimize the size of the quantum register, we use the following formulas to determine the number of qubits $\lambda =\lceil \log_{2}\left(I+1\right)\rceil$ and $\mu =\lceil \log_{2}\left(J+1\right)\rceil$. Each qubit has an index within this register, starting at index 0 and counting up by 1 till *M*. Besides this, we use λ qubits (instead of λV) for the determination placement of virtual machines because of a key advantage of the quantum register. It can proceed with $2^{\lambda }$ virtual machines placements, concurrently. We can create digital decision variables by using a roulette wheel due to the given probability distribution after the measurement of the quantum register (Figure 1). Similarly, we reduce the number of qubits from μI to μ, allowing for the allocation of appropriate hosts to VMs clusters. Therefore, the quantum register consists of *M* = λ+μ, only.

An outcome of measuring is saved into a binary measurement register *BMQ* with the same number of entries as the qubit register. Declared binary states of *BMQ* entries are 0 or 1. When a qubit of the register *Q* is measured the second time, the corresponding bit in the binary register is overwritten by the new measurement bit, even when a measurement is done on a basis different than the basis used for an earlier measurement. In this case, the selection of *x*-basis, *z*-basis or *z*-basis does not allow the storage of the previously measured bit in the register *BMQ*. The most recent qubit measurement introduces achange to the associated binary bit of the measurement register. The quantum register *Q* can be measured regarding the *z*-basis of each qubit. Figure 1 shows an example of a probability distribution for placement of the *v*th virtual machine. This distribution is important to generate digital positions of particles. 

An initial population *P*(*t*) of *L* particles *px*(*t*) *=* (*x*(*t*), *v*(*t*)) is created by measuring the state of the register *Q* at the iteration *t* (Algorithm 1, step 4). The current position at the iteration *t* is encoded as $x\left(t\right)=\left[X_{1}^{\alpha }\left(t\right),\ldots ,X_{v}^{\alpha }\left(t\right),\ldots ,X_{V}^{\alpha }\left(t\right),X_{1}^{\beta }\left(t\right),\ldots ,X_{i}^{\beta }\left(t\right),\ldots X_{I}^{\beta }\left(t\right)\right].$ Besides, the velocity $0\leq v\left(t\right)\leq v_{max}$ of this particle has *V* + *I* coordinates, too. Therefore, the digital particle px(t) has 2(*V* + *I*) coordinates. Placements of virtual machines are randomly selected *V* times due to the roulette wheel constructed by the probability distribution provided by the quantum register *Q* (Figure 1). Also, the hosts with adequate resources to clusters of virtual machines are randomly chosen *I* times by the roulette wheel related to measuring another part of the quantum register *Q*. Besides this, the velocity vector v of this digital particle is created by generation *V* + *I* values for $0\leq v_{m}^{}\left(t\right)\leq v^{max}$. Based on the quantum register *Q*, *L* digital positions of particles can be created to establish the initial population *P*(*t* = 0), where *px*(*t*) = (*x*(*t*), *v*(*t*)) and *L* is the size of a population. 

**Figure 2:**
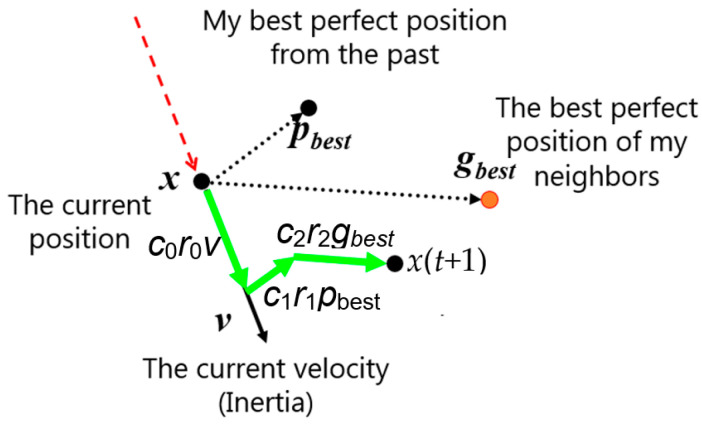
Determination of the new position of a digital particle.

The population *P*(*t*) enables the designation of an offspring population *B*(*t*) in accordance with the rules of canonical PSO algorithms (Algorithm 1, step 5). The new position is calculated by adding three vectors to the current position *x*(*t*). The first vector is a difference between the best position *p_best_* of this particle from the past and the current position. The vector is multiplied by a random number *r*_1_ from the interval [0; 1] and by the given coefficient *c*_1_. The second vector is a difference between the best perfect position *g_best_* of the neighborhood and the current position. This vector is multiplied by a random number *r*_2_ from the interval [0; 1] and by the given coefficient *c*_2_. The third vector is the difference between the velocity and the current position. This vector is multiplied by a random number *r*_0_ from the interval [0; 1] and by the given coefficient *c*_0_ (Figure 2). 

An extended archive *C*(*t*) is the sum of three sets of particles *A*(*t*)∪*B*(*t*)∪*P*(*t*) (Algorithm 1, step 6). We compare particles from the extended archive *C*(*t*). Criteria values of particles are calculated, followed by Fonseca–Fleming ranks [36]. A rank *r*(*x*) of solution *x* is a number of dominant solutions from *C*(*t*). 

The next step of the algorithm MQPSO is calculation crowding distances, fitness values, and then sorting particles in *C*(*t*) (Algorithm 1, step 7). Each particle is characterized by crowding distance to determine its fitness and to distinguish solutions with the same rank [37]. Sorted particles with the highest fitness values are qualified to an archive *A*(t) of non-dominated solutions with their criteria values (Algorithm 1, step 8). 

An important step of an algorithm is using three rotation gates to modify the quantum register *Q* (Algorithm 1, steps 9–11). The $R_{x}$ gate is a single-qubit rotation through the angle $\theta_{x}$ (radians) around the *x*-axis. Similarly, the $R_{y}$ gate is a rotation through the angle $\theta_{y}$ around the *y*-axis. A rotation through $\theta_{z}$ around the *z*-axis is the $R_{z}$ gate. The adequate matrix operations can be written, as follows [35]:

(30) $$R_{x}\left(\theta_{x}\right)=\left[\begin{array}{cc} \cos\frac{\theta_{x}}{2} & -i\sin\frac{\theta_{x}}{2}\\ -i\sin\frac{\theta_{x}}{2} & \cos\frac{\theta_{x}}{2} \end{array}\right]$$

(31) $$R_{y}\left(\theta_{y}\right)=\left[\begin{array}{cc} \cos\frac{\theta_{y}}{2} & -\sin\frac{\theta_{y}}{2}\\ \sin\frac{\theta_{y}}{2} & \cos\frac{\theta_{y}}{2} \end{array}\right]$$

(32) $$R_{z}\left(\theta_{z}\right)=\left[\begin{array}{cc} e^{-i\frac{\theta_{z}}{2}} & 0\\ 0 & e^{i\frac{\theta_{z}}{2}} \end{array}\right]$$

**Figure 3:**
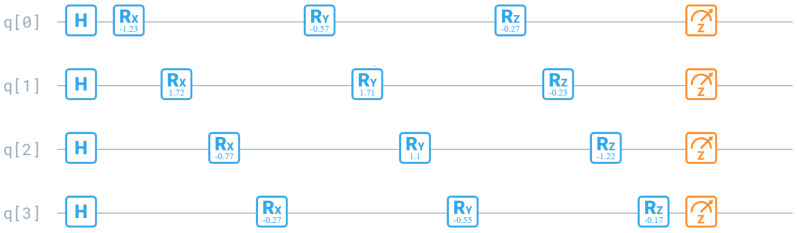
Hadamard and rotation gates for updating the quantum register *Q*.

Figure 3 shows the quantum gates for finding the correction of new particle position. It determines the new assignment of the *v*th virtual machine to the host. There are Hadamard gates and three rotation gates that determine host number. An important role play rotation angles $\theta_{x},\;\theta_{y},\;\theta_{z}$ for each qubit $m=\bar{1,M}$. A matrix of angles $M_{\theta }$ can be characterized, as below:

(33) $$M_{\theta }=\left[\begin{array}{ccc} \theta_{x1}\;\ldots & \theta_{xm}\;\ldots & \theta_{xM}\\ \theta_{y1\;}\ldots & \theta_{ym}\;\ldots & \theta_{yM}\\ \theta_{z1}\;\ldots & \theta_{zm}\;\ldots & \theta_{zM} \end{array}\right]$$

Initially, the angles are determined randomly. However, preferences should be given to modifications of the quantum register, which cause greater effects in the set of designated non-dominated solutions in the archive. For this reason, we evaluate each rotational angle matrix with the number of effective solutions in the archive, which solutions were determined using a given matrix. A matrix $M_{\theta }$ with a higher rating $R\left(M_{\theta }\right)$ is more likely to be used in the next iteration because of a tournament selection of the rotation angle matrix in conjunction with the roulette rule. Each angle of the matrix can be mutated at the *p_m_* rate, which mutation consists in changing the angle by a random value with a normal distribution with standard deviation σ.

**Figure 4:**
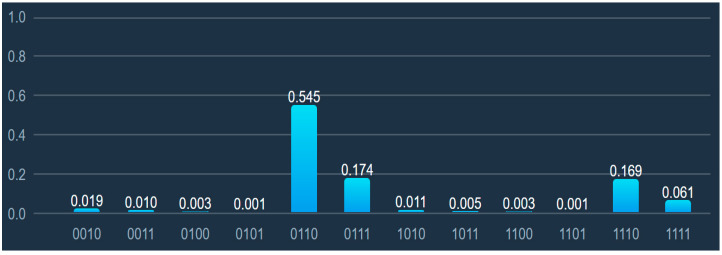
Distribution of the node selection probabilities after rotations of quantum register.

Figure 4 shows results after rotations the quantum register *Q* followed by measurement. The most preferred host by virtual machines is located at the sixth node. Placement both at the 7th and the 15th node have much fewer chances to be selected, but they may be chosen for several VMs.

To sum up, the population *P*(*t*) of *L* particles is created by observing the state of the quantum register *Q*(*t*) in main loop of MQPSO. New positions and velocities of particles are generated followed by create a neighborhood *B*(*t*). Besides, values of criteria are calculated, and solutions are verified if they satisfy constraints. Then, we can find ranks of feasible solutions for an extended archive *C*(*t*) = *A*(*t −* 1)∪*B*(*t*)∪*P*(*t*). If a rank is equal to zero, a solution is non-dominated in an extended archive. 

Non-dominated solutions are accepted for the *A*(*t*) archive, only. If the number of Pareto-optimal solutions exceeds the archive size, a representation of them is qualified, which takes place by means of the densification function. Solutions with ratings in less dense areas have a greater chance of qualifying for the archive. In the initial period of searching the space of feasible solutions, solutions with higher ranks and even unacceptable solutions based on the fitness function may be qualified.

The algorithm ends the exploration of space when the time limit is exceeded (the number of particle population generations) or when there is no improvement over a given number of iterations.

## 6. Pareto-Optimal Solutions and Compromise Alternatives

Let $X_{n}^{Pareto}$ be a set of Pareto-optimal solutions for the many-objective problem of virtual machine placement $(X,\;F,\;{⥼}_{\leq }$) (18)–(23) with *n* criteria, where *n* = 2, 3, …, 7. The set X of admissible solutions is the same for each *n*. Because there are seven partial criteria ${\hat{Z}}_{max}$, ${\tilde{Z}}_{max}$, $\Xi ,\;E,-\kappa_{min}^{RAM},-\kappa_{min}^{disc},-R$, we can use a notation $F=\left[F_{1},\;\ldots ,F_{n},\ldots ,F_{N=7}\right].$ There are six sets of Pareto solutions: $X_{2}^{Pareto},\;X_{3}^{Pareto},\;\ldots ,\;X_{7}^{Pareto}$ because n=2, 3,…, 7. Also, there are six sets of evaluations Y=F(X). 

Besides this, *n* dimensional domination relation in $R^{n}$ denoted as ${⥼}_{n}$ can be defined, as below:

(34) $${⥼}_{n}=\left\{\left(a,\;b\right)\in Y\times Y|a_{i}\leq b_{i},\;i=\bar{1,n}\right\},\;Y=F\left(X\right)\subset R^{n},\;n=\bar{2,7}$$

We can formally explain the number growth of Pareto-optimal solutions in many-objective optimization problems due to adding the partial criteria by the following theorem.

**Theorem** **1.** *A set of Pareto solutions $X_{n}^{Pareto}\subseteq X$ for n (n* *≥ 2) criteria in the many-criteria optimization problem of virtual machines placement $(X,\;F,\;{⥼}_{\leq }$) (18)–(23) is included in the set of Pareto solutions $X_{n+k}^{Pareto}\subseteq X$ for n + k criteria, k = 1,2, …, N − n (n + k ≤ N) and a domination relation ${⥼}_{n}$ in $R^{n+k}$, which can be formulated, as below:*

(35) $$X_{n}^{Pareto}\subseteq X_{n+k}^{Pareto},\;k=1,2,\ldots ,N-n\;\;$$

**Proof.**  Let $X_{2}^{Pareto}$ be a non-empty set of Pareto solutions for two criteria *F*_1_ and *F*_2_. If we add the third criterion *F*_3_, all solutions from $X_{2}^{Pareto}$ are still Pareto-optimal. Besides, there is no admissible solution $x\in X,\;x\notin X_{2}^{Pareto}$ that dominates all solutions from the set $X_{2}^{Pareto}$ due to three criteria $F_{1},\;F_{2},F_{3}$. On the other hand, another non-dominated solution $x\in X,\;x\notin X_{2}^{Pareto}$ may exist regarding a smaller value of $F_{3}\left(x\right)$. Therefore, $X_{2}^{Pareto}\subseteq X_{3}^{Pareto}$. Also, we can proof $X_{3}^{Pareto}\subseteq X_{4}^{Pareto}$, $X_{4}^{Pareto}\subseteq X_{5}^{Pareto}$ and so on.

We have shown that for every natural number *k* ≥ 1 the implication *T*(*k*) ⇒ *T*(*k* + 1) is true since the truth of its predecessor implies the truth of the successor. Since the assumptions of the mathematical induction rule are satisfied for this theorem, the formula (35) is true for every *k* ≥ 1, which ends the proof. □

It can happen that $X_{n}^{Pareto}=X_{n+k}^{Pareto}$, but this is extremely rare. Usually, along with additional criteria, the size of the Pareto set increases significantly in the many-criteria optimization problem of virtual machines placement $(X,\;F,\;{⥼}_{\leq }$) (18)–(23). 

**Figure 5:**
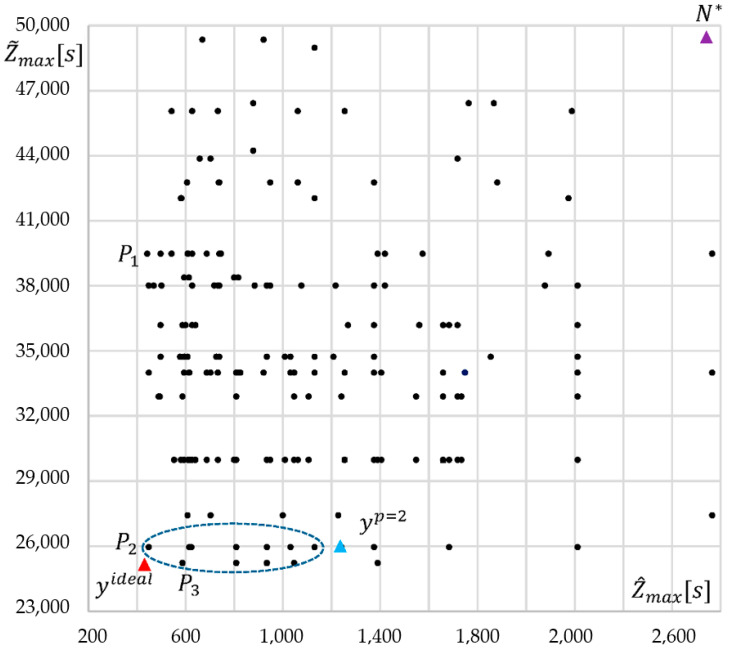
Pareto-optimal evaluations of two criteria $({\hat{Z}}_{max}$, ${\tilde{Z}}_{max})$ for Benchmark855.

The algorithm MQPSO determined the compromise solution (Figure 5) characterized by the score $y^{p=2}$ = (1240; 25,952; 10,244; 11,630; 18; 191; 0.92) with the smallest Euclidean distance to an ideal point $y^{ideal}$ = (442; 25,221; 6942; 6750; 19; 195; 0.97) in the normalized criterion space $\bar{Y}$. Coordinates of an ideal point are calculated regarding the following formulas:

(36) $$y_{n}^{ideal}=\{\begin{array}{c} \underset{x\in X_{7}^{Pareto}}{\min}F_{n}\left(x\right),\;n=\bar{1,4}\\ \underset{x\in X_{7}^{Pareto}}{\max}F_{n}\left(x\right),\;n=\bar{5,7} \end{array}$$

The nadir point $N^{*}$ is another characteristic point of the criterion space Y**.** The nadir point is required to normalize the criterion space. Contrary to the ideal point, $N^{*}$ takes into account the worst values of the Pareto set $Y_{7}^{Pareto}=F\left(X_{7}^{Pareto}\right)$ in terms of the preferences, as follows:

(37) $$N_{n}^{*}=\{\begin{array}{c} \underset{x\in X_{7}^{Pareto}}{\max}F_{n}\left(x\right),\;n=\bar{1,4}\\ \underset{x\in X_{7}^{Pareto}}{\min}F_{n}\left(x\right),\;n=\bar{5,7} \end{array}$$

Moreover, an anti-ideal point $P_{n}^{sup}$ may be used for the normalization of a criterion space. Coordinates of an anti-ideal point are calculated due to the following formulas:

(38) $$P_{n}^{sup}=\{\begin{array}{c} \underset{x\in X}{\max}F_{n}\left(x\right),\;n=\bar{1,4}\\ \underset{x\in X}{\min}F_{n}\left(x\right),\;n=\bar{5,7} \end{array}$$

Because an algorithm determines the Pareto set of solutions $X_{7}^{Pareto}$ and its evaluation set $Y_{7}^{Pareto}=F\left(X_{7}^{Pareto}\right)$, we can normalize an evaluation set $Y_{7}^{Pareto}$ as the 7D hypercube ${\bar{Y}}_{7}^{Pareto}=$ [0; 1]^7^, where the normalized ideal point is ${\bar{y}}^{ideal}$ = (0; 0; 0; 0; 1; 1; 1), as below:

(39) $${\bar{y}}_{n}^{}=\{\begin{array}{c} \frac{F_{n}\left(x\right)-F_{n}^{ideal}}{N_{n}^{*}-F_{n}^{ideal}},\;n=\bar{1,4}\\ \frac{F_{n}\left(x\right)-N_{n}^{*}}{F_{n}^{ideal}-N_{n}^{*}},\;n=\bar{5,7} \end{array}$$

The normalized nadir point ${\bar{N}}^{*}$ = (1; 1; 1; 1; 0; 0; 0) is characterized by the maximum Euclidean distance $\sqrt{7}\approx 2.65$ from the normalized ideal point. In the hypercube ${\bar{Y}}_{7}^{Pareto}$, a trade-off (compromise) placement of virtual machines $\omega^{p}$ can be selected from the Pareto-optimal set $X_{7}^{Pareto}$ due to the smallest value of *p-norm* $L_{p}$, as follows:

(40) $$L_{p}\left({\bar{y}}^{p}\right)=\underset{\bar{y}\in {\bar{Y}}_{7}^{Pareto}}{\min}L_{p}\left(\bar{y}\right),\;p=1,2,\ldots$$

where

${\bar{y}}^{p}$ is the normalization evaluation point of $y^{p}=F\left(\omega^{p}\right)\in Y_{7}^{Pareto}$,
$L_{p}\left(\bar{y}\right)={\left\|\bar{y}-{\bar{y}}^{ideal}\right\|}_{p}={\left(\sum_{n=1}^{7}{\left(\bar{y_{n}}-{\bar{y_{n}}}^{ideal}\right)}^{p}\right)}^{1/p}$.

**Theorem** **2.** *For the given parameter p = 1, p = 2 or p→∞, the normalization (39) and a domination relation* ${⥼}_{\leq }$ *in the many-criteria optimization problem of virtual machines placement* $(X,\;F,\;{⥼}_{\leq }$ *) (18)–(23), p-norm* $L_{p}$ *is a function of solution x, as follows:*

(41) $$\;L_{1}\left(x\right)=\sum_{n=1}^{4}\frac{F_{n}\left(x\right)-F_{n}^{ideal}}{N_{n}^{*}-F_{n}^{ideal}}+\sum_{n=5}^{7}\frac{F_{n}\left(x\right)-N_{n}^{*}}{F_{n}^{ideal}-N_{n}^{*}}\;\;$$

(42) $$\;L_{2}\left(x\right)=\sqrt{\sum_{n=1}^{4}{\left(\frac{F_{n}\left(x\right)-F_{n}^{ideal}}{N_{n}^{*}-F_{n}^{ideal}}\right)}^{2}+\sum_{n=5}^{7}{\left(\frac{F_{n}\left(x\right)-N_{n}^{*}}{F_{n}^{ideal}-N_{n}^{*}}\right)}^{2}}\;\;$$

(43) $$\;L_{\infty }\left(x\right)=\underset{}{\max}\left\{\underset{n=\bar{1,4}}{\max}\frac{F_{n}\left(x\right)-F_{n}^{ideal}}{N_{n}^{*}-F_{n}^{ideal}},\underset{n=\bar{5,7}}{\max}\frac{F_{n}\left(x\right)-N_{n}^{*}}{F_{n}^{ideal}-N_{n}^{*}}\right\}\;\;$$

**Proof.**  Let *p* = 1. Then, $L_{1}\left(\bar{y}\right)={\left\|\bar{y}-{\bar{y}}^{ideal}\right\|}_{1}={\left(\sum_{n=1}^{7}{\left(\bar{y_{n}}-{\bar{y_{n}}}^{ideal}\right)}^{1}\right)}^{1/1}=\sum_{n=1}^{7}{\left(\bar{y_{n}}-{\bar{y_{n}}}^{ideal}\right)}^{}$. Because ${\bar{y}}^{ideal}$ = (0; 0; 0; 0; 1; 1; 1), we get $L_{1}\left(\bar{y}\right)=\sum_{n=1}^{4}{\bar{y}}_{n}+\sum_{n=5}^{7}\left({\bar{y}}_{n}-1\right)$. We insert the right side of Equation (39) instead of ${\bar{y}}_{n}$, and we get the Equation (41). We prove the correctness of formulas (42) and (43) in a similar way, which ends the proof. □

## 7. Numerical Experiments

In order to verify the quality of the mathematical model, the correctness of the formulated many-criteria optimization problem, as well as the quality of the developed algorithm, several multi-variant numerical experiments were carried out, and the designated compromise solutions were simulated in the GUT-WUT cloud computing environment. We consider four instances of the virtual machine placement problem such as: Benchmark90, Benchmark306, Benchmark855 and Benchmark1020 that are available on site https://www.researchgate.net/profile/Piotr-Dryja (accessed: 19 November 2022). For example, Benchmark855 is characterized by 855 binary decision variables, and therefore a binary search space contains 2.4 × 10^257^ items. There are 45 VMs, 15 nodes and 12 possible hosts. Besides this, there are both 60 integer decision variables and 1.3 × 10^69^ possible solutions. 

If we consider seven criteria, there are 21 pairs: $\left({\hat{Z}}_{max},{\tilde{Z}}_{max}\right),\;\left({\hat{Z}}_{max},\Xi \right),\;\left({\hat{Z}}_{max},E\right),\;\left({\hat{Z}}_{max},\kappa_{min}^{RAM}\right),\;\left({\hat{Z}}_{max},\kappa_{min}^{disc}\right),\;\left({\hat{Z}}_{max},R\right),$ (${\tilde{Z}}_{max},\;\Xi )$ and so on. Figure 5 shows three evaluations of Pareto-optimal solutions in two criteria spaces $\left({\hat{Z}}_{max},{\tilde{Z}}_{max}\right).$ Points P_1_ = (410; 395,223), P_2_ = (448; 25,952) and P_3_ = (587; 25,221) are non-dominated due to ${\hat{Z}}_{max}$ $\mathrm{and}\;{\tilde{Z}}_{max}$. However, the results of the experiments confirmed a significant increase in the number of Pareto-optimal solutions with the addition of further criteria. For instance, the other criteria $\Xi ,\;E,\;\kappa_{min}^{RAM},\kappa_{min}^{disc},R$ significantly extended the set of Pareto solutions to a set {P_1_, P_2_, …, P_200_}. While these supplementary 197 points are dominated by two criteria ${\hat{Z}}_{max}$, ${\tilde{Z}}_{max}$, each new criterion usually increases the number of Pareto-optimal solutions that dominate points P_1_, P_2_, P_3_ due to this new metric. As a result, we can expect several Pareto-optimal solutions from which we can choose the compromise evaluation $y^{p=2}$ = (1240; 25,952; 10,244; 11,630; 18; 191; 0.92), where each evaluation of solution is presented as *y*(*x*) = $\left({\hat{Z}}_{max}\left(x\right),\;{\tilde{Z}}_{max}\left(x\right),\;\Xi \left(x\right),\;E\left(x\right),\kappa_{min}^{RAM}\left(x\right),\kappa_{min}^{disc}\left(x\right),R\left(x\right)\right)$. The trade-off evaluation $y^{p=2}=F\left(x^{p=2}\right)$ minimizes Euclidean distance to an ideal point in the normalized space R^7^. On the other hand, P_2_ is the compromise point in the normalized space **R**^2^.

**Figure 6:**
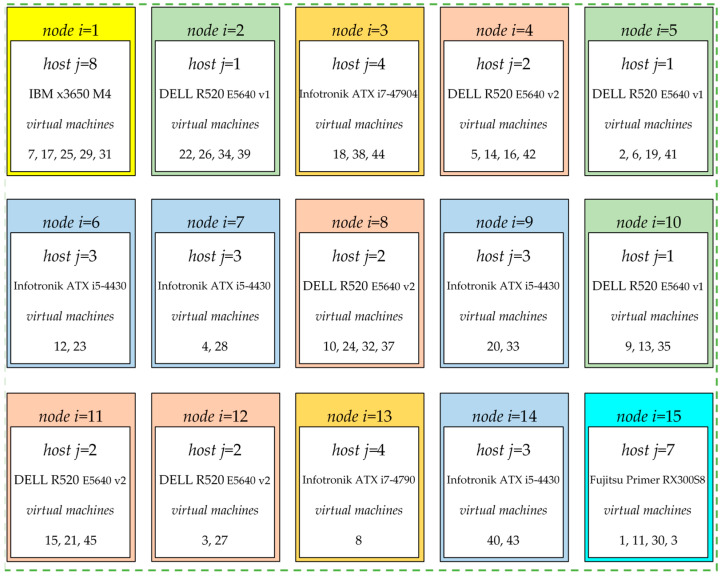
The compromise placement of 45 VMs $x^{p=2}$ for Benchmark855.

Figure 6 shows the compromise placement of virtual machines. A solution $x^{p=2}$ specifies 15 destinations for 45 virtual machines, where the adequate resources are provided to efficient run all tasks. There are three hosts DELL R520 E5640 v1 (Dell Inc., USA), 4 DELL R520 E5640 v2, 4 Infotronik ATX i5-4430 (Infotronik, Poland), 2 Infotronik ATX i7-4790, Fujitsu Primergy RX300S8 (Fujitsu, Japan) and IBM x3650 M4 (IBM, USA) allocated at 15 nodes.

**Figure 7:**
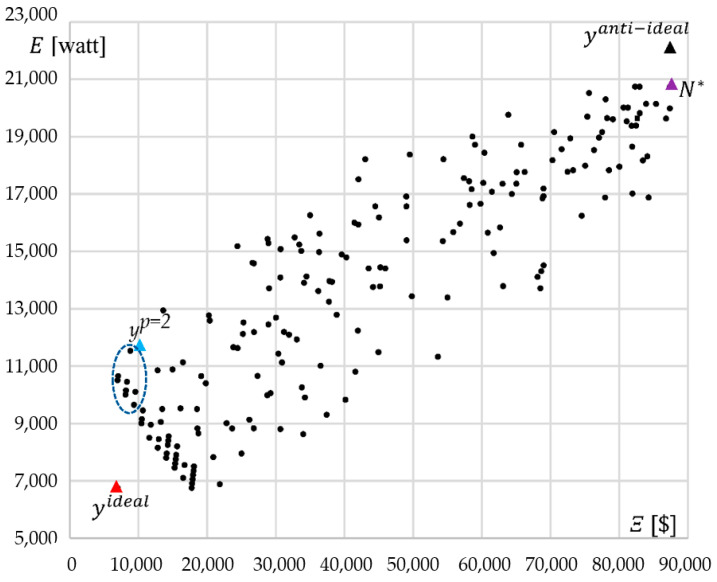
Pareto-optimal evaluations for two selected criteria Ξ and E.

The 7D compromise estimation $y^{p=2}=F\left(x^{p=2}\right)$ is dominated by the other solutions due to several pairs of criteria, but there is at least one pair of criteria, where it is non-dominated. Figure 7 shows Pareto evaluations found by MQPSO for the cut (Ξ, E). In this case, the compromise point is dominated by seven evaluation points. However, $y^{p=2}$ is close to the Pareto front of this pair criterion cut (Ξ, E). A similar situation occurs in Figure 5, where the compromise score is dominated by 11 elements. On the other side, these evaluations are dominated by the compromise solution in Figure 6. In summary, the compromise solution is not dominated by other alternatives in the sense of the four criteria and is, therefore, not dominated in the sense of the seven criteria as well.

The 7D estimation $y^{p=2}$ of compromise solution was determined for an ideal point $y^{ideal}$ = (442; 25,221; 6942; 6750; 19; 195; 0.97) (Table 1). For normalization of the criterion space, the nadir point N* = (2764; 49,346; 87,359; 20,740; 5.2; 21.5; 0.53) was used. Besides this, it was calculated the anti-ideal point $y^{anti-ideal}$ (3600; 50,000; 87,500; 22,000; 4; 7; 0.33) that can be applied for an alternative normalization. A selection of a compromise solution is carried out in the normalized space due to minimization *p*-norm $L_{p}$.

If we choose the nadir point N* for the normalization of the criterion space ***Y***, the evaluation of the compromise solution is $y^{p=2}$ for *p* = 1 and *p* = 2 (No. 1 in Table 1). Without losing the generality of the considerations, Table 1 presents the best 20 solutions in the sense of $L_{2}$ and normalization using the nadir point. When analyzing the coordinates of the points closest to the ideal point, it can be noticed that in this case the “middle” values of the criteria are preferred instead of lexicographic solutions, which are characterized by the best value of the selected criterion. The most preferred values in each of the seven categories are marked in yellow (Table 1).

The undoubted advantage of the compromise solution is its full dominance over other competitors due to the size of the disk storage reserve in the most critical host. In this respect, the remaining solutions are characterized by slightly worse values. The advantage is also the largest reserve of RAM memory because only solution number 4 (Table 1) has the same value. Moreover, the compromise alternative has the shortest data transmission time through the busiest cloud transmission node. In this case, solutions No. 5, 9 and 18 are also characterized by an equally high quality of data transmission. Consuming more electricity than several solutions is perhaps the biggest disadvantage of the compromise solution. However, this is not too much of a difference to the most energy-efficient placements of the VMs migration.

Solutions No. 2 and 3 are characterized by lower electric power consumption by more than 2 kilowatts. Furthermore, they are not the best in terms of any criterion, but in terms of $L_{2}$, they are very close to the compromise solution. 

If we choose the nadir point N* for the normalization, the evaluation of compromise solution is $y^{p\to \infty }$ for *p*→∞ (No. 4 in Table 1). Table 2 presents the *p*-norm values for the best 20 Pareto-optimal VMs placements sorted by $L_{2}$. Solution No. 4 differs from $x^{p=2}$ in that all criteria values are more balanced with respect to the ideal point coordinates. This is due to the greater consumption of electricity by the solution No. 1, which causes the value of the $L_{p\to \infty }$ to be 0.349. On the other hand, the solution No. 4 is characterized by $L_{p\to \infty }=$ 0.335.

If we select the anti-ideal point $y^{anti-ideal}$, the differences between the coordinate values of the point and the ideal point are greater than when the nadir point is taken into account. As a result, we are dealing with a completely different normalization. The specificity of this computational instance is such that the ideal point has the greatest impact on the change of normalization for the reliability of the cloud because the distance to the ideal point coordinate is increased by 45.5%. On the other hand, the increase in the length of the value range of 36% is characterized by the load of the CPU bottleneck host in the computing cloud. For the other five criteria, the impact on standardization is below 10%. 

However, the change of the normalization point with respect to the ideal point did not result in any major changes related to the compromise solutions. Solution No. 1 remained a compromise solution for *p* = 1 and for *p* = 2. On the other hand, a new compromise solution has been identified for *p*→∞ (Table 1, No. 6). In this case, $\Delta \bar{y_{1}}$ decreased from 0.350 to 0.260, which caused $L_{p\to \infty }$ to be affected by $\Delta \bar{y_{4}}$, which is 0.290.

The decision-maker can choose one value of the parameter *p*. We prefer an influence of all criteria on the compromise solutions for *p* = 1 but some of them can achieve very poor values. On the other hand, if *p* = 2, we favor the minimal Euclidean distance to the ideal point. Finally, all criteria achieve similar good values not far from ideal ones if *p*→∞ is selected. 

Another dilemma is related to a selection between the nadir point and the anti-ideal point for the normalization of the criterion space. The nadir point gives information about a range of all efficient solutions. Besides this, the anti-ideal point gives information about the range of the admissible set. If the selection of compromise solutions is considered from the Pareto set, the nadir point is more suitable than the anti-ideal point for the criterion space normalization. As a consequence, the compromise solution can be selected. 

We suggest selecting both *p* = 2 and the nadir point to determine the compromise solution from the set of Pareto-optimal elements of two or three criteria. The other approach is based on many-objective analysis with seven criteria, where an extended analysis is needed because of greater sensitivity of compromise solutions to the parameter *p* and the choice of the normalization point. In this way, a decision-maker can find some trade-off solutions after introducing a limit on the size of the representation of Pareto-optimal solutions, which is the specificity of solving optimization problems with many criteria.

A very important experiment is to compare the quality of the designated solutions by the proposed method with other methods. Outcome evaluations of Pareto placement of virtual machines are presented in Table 3. The Benchmark855 was used for this purpose, too. We consider fifteen non-dominated solutions obtained by MQPSO, Non-dominated Sorting Genetic Algorithm II (NSGA-II) [37], Multi-criteria Genetic Programming (MGP) [38], Multi-criteria Differential Evolution (MDE) [4] and Multi-criteria Harmony Search (MHS) [39]. 

To compare 15 solutions provided by five metaheuristics, we achieved a new ideal point $y^{ideal}$ = (385; 2193; 10,242; 9500; 18; 191; 0.92). In addition, the new nadir point N* = (1405; 36,187; 33,259; 14,838; 11; 112; 0.83) was used for normalization. When analyzing the computational load of each algorithm, it was assumed that the population consists of 100 particles (MQPSO), chromosomes (MDE, MHS) or compact programs (MGP). The population number is set to 10,000 and the maximum computation time is 30 min. In MQPSO, the values of individual coefficients were as follows: *c*_0_ = 1, *c*_1_ = 2, *c*_2_ = 2, *v*_max_ = 1. In the differential evolution algorithm MSE, *q* = 0.9 and *Cp* = 0.4 were assumed. Moreover, an additional type of mutation was used, based on the multi-criteria tabu search algorithm [2]. In contrast, in the harmony search MHS algorithm, the mutation rate p_m_ was 0.1 and the crossover rate *p_c_* was 0.01. Using MGP genetic programming, it was assumed that the maximum number of nodes in the programming tree was 50, and the mutation rate and crossing rate were the same as for MHS. 

An optimal swarm size is problem-dependent. If the number of particles in the swarm is greater, the initial diversity is larger, and a larger search space is explored. On the other hand, more particles increase the computational complexity, and the PSO exploration leads to a parallel random search. We observed that more particles lead to fewer swarms to reach the Pareto-optimal solutions, compared to a smaller number of particles. Our experiments confirmed that the MQPSO has the ability to find Pareto-optimal solutions with sizes of 60 to 100 particles. Each run was repeated 10 times, and Table 3 lists the best solutions obtained with each algorithm. The *p*-norm values for the best 15 Pareto-optimal VMs placements were sorted by *L*_2_. Moreover, the other values for *p*-norm were calculated, too. 

Based on the obtained data, it can be concluded that the MQPSO method is better than the other methods due to the number of Pareto-optimal solutions in the first 12. The closest three solutions to the ideal point are determined with MQPSO. An important argument is also the fact that the average distance from the ideal point is the smallest for effective solutions provided by the MQPSO method. NSGA-II is the second metaheuristic with an average distance of 1.39 versus 1.16 achieved by MQPSO. If we consider the *p*-norm for *p* = 1, the compromise solution is the same. Also, the three nearest solutions to the ideal point are produced by MQPSO. On the other hand, solution No. 10 provided by NSGA-II and solution No. 14 determined by MDE are very close to the compromise solution due to ***L_p_*_→__∞_**. Moreover, the algorithm MQPSO has great potential to be extended in the nearest feature due to development of the quantum computers and a quantum algorithmic theory.

## 8. Concluding Remarks

Smart education systems, intelligent health care and smart cities require deep learning models and efficient management of computer resources that can be supported by the live migration of virtual machines. Because the formulated problem of many-objective optimization is NP-hard, we proposed the many-objective PSO algorithm with quantum gates to provide Pareto-optimal placements of virtual machines in computing clouds. Efficient solutions determined by MQPSO satisfy seven criteria such as electric power of hosts, reliability of the cloud, the workload of the bottleneck host, communication capacity of the critical node, RAM usage, disc memory capacity and computer costs. Hadamard gates support forming an initial population in the quantum register by introducing a superposition of qubits. Also, rotation gates can change the current state of the quantum register to explore the neighborhood of the current particle. Extensive numerical results from the experimental cloud based on the OpenStack platform showed that MQPSO is a very efficient tool supporting the management of live migration in the computing cloud.

The cloud can share the workload, which permits efficient training of machine learning algorithms, too. Solvers based on MQPSO can find the compromise solution for parameter *p* = 2 from the set of Pareto-optimal alternatives that is a recommendation regarding teleportation of virtual machines. Due to the experimental validation of Pareto solutions, a higher quality performance of the cloud is achieved than the performance obtained by solutions from well-known algorithms such as genetic programming or differential evolution.

In our future work, we are going to study the other metaheuristics with quantum gates for the migration of virtual machines with the extended set of optimization criteria. 

## Figures and Tables

**Table 1:** Criteria values for the best 20 Pareto-optimal solutions sorted by *L*_2_ in the normalized criterion space by the nadir point.

No.	${\hat{Z}}_{max}(s)$	${\tilde{Z}}_{max}(s)$	Ξ ($)	*E* (watt)	$\kappa_{min}^{RAM}\;\left(\mathrm{GB}\right)$	$\kappa_{min}^{disc}(\mathrm{TB})$	R
1	1240.78	25,952.49	10,244.00	11,630.00	18.0	191	0.92
2	1404.84	29,973.30	13,455.00	9500.00	17.5	190	0.90
3	1389.77	29,973.30	16,114.00	9525.00	17.0	185	0.91
4	1105.84	29,973.30	30,352.00	11,430.00	18.0	188	0.91
5	1240.78	25,952.49	24,412.00	11,625.00	16.0	180	0.92
6	1254.24	29,973.30	30,895.00	11,125.00	17.0	190	0.91
7	1061.17	29,973.30	20,240.00	12,760.00	17.0	165	0.88
8	1374.70	29,973.30	8796.00	11,530.00	15.0	187	0.88
9	1683.01	25,952.49	20,888.00	7825.00	14.0	188	0.87
10	1240.78	32,897.52	19,126.00	10,650.00	16.0	188	0.78
11	1733.44	29,973.30	15,623.00	8200.00	17.0	98	0.88
12	1547.61	29,973.30	8,317.00	10,450.00	12.0	122	0.90
13	1683.01	29,973.30	9329.00	9650.00	14.0	132	0.77
14	1718.37	29,973.30	14,248.00	8250.00	11.0	123	0.88
15	1683.01	29,973.30	14,393.00	8550.00	12.0	122	0.77
16	1733.44	29,973.30	12,945.00	8450.00	15.0	99	0.75
17	1658.75	29,973.30	18,574.00	8825.00	8.0	155	0.97
18	1683.01	25,952.49	18,509.00	9500.00	9.0	77	0.89
19	1061.17	29,973.30	36,498.00	11,010.00	17.0	21	0.77
20	1130.09	34,725.16	29,234.00	10,055.00	12.0	98	0.59

**Table 2:** The *p*-norm values for the best 20 Pareto-optimal VMs placements sorted by *L*_2_.

No.	*L_p_* for the Nadir Point			*L_p_* for the Anti-Ideal Point		
	*p* = 1	*p* = 2	*p*→∞	*p* = 1	*p* = 2	*p*→∞
1	0.973	0.511	0.349	0.810	0.424	0.320
2	1.186	0.542	0.415	0.994	0.438	0.305
3	1.256	0.548	0.408	1.068	0.450	0.300
4	1.358	0.585	0.335	1.197	0.524	0.307
5	1.358	0.596	0.348	1.177	0.516	0.320
6	1.467	0.623	0.350	1.287	0.549	0.297
7	1.581	0.644	0.430	1.381	0.566	0.394
8	1.504	0.667	0.402	1.273	0.562	0.313
9	1.445	0.712	0.534	1.193	0.572	0.393
10	1.782	0.745	0.432	1.504	0.615	0.310
11	1.873	0.864	0.559	1.593	0.727	0.516
12	2.042	0.892	0.507	1.766	0.774	0.467
13	2.148	0.915	0.534	1.785	0.741	0.393
14	2.144	0.954	0.580	1.842	0.818	0.533
15	2.335	0.995	0.534	1.963	0.825	0.467
16	2.292	1.004	0.556	1.908	0.820	0.511
17	2.042	1.022	0.797	1.804	0.899	0.733
18	2.492	1.169	0.725	2.166	1.031	0.667
19	2.738	1.253	1.000	2.405	1.122	0.926
20	3777.134	1.301	0.864	2.671	1.075	0.594

**Table 3:** Criteria values for 15 Pareto-optimal solutions determined by five multi-objective metaheuristics.

No.	Algorithm	${\hat{Z}}_{max}(s)$	${\tilde{Z}}_{max}(s)$	Ξ ($)	*E* (watt)	$\kappa_{min}^{RAM}\;$ (GB)	$\kappa_{min}^{disc}(\mathrm{TB})$	R	*L*_1_	*L_2_*	*L_p_*_→__∞_
1	MQPSO	1241	25,952	10,244	11,630	18	191	0.92	1.52	0.97	0.84
2	MQPSO	1405	29,973	13,455	9500	18	190	0.9	2.01	1.18	1.00
3	MQPSO	1390	29,973	16,114	9525	17	185	0.91	2.14	1.18	0.99
4	NSGA-II	587	25,221	33,259	12,223	15	177	0.92	2.54	1.25	1.00
5	NSGA-II	442	25,221	12,133	12,163	17	112	0.87	2.57	1.28	1.00
6	MDE	442	29,973	15,351	12,675	16	152	0.84	2.89	1.29	0.89
7	MDE	581	25,221	15,292	11,598	11	149	0.89	2.90	1.30	1.00
8	MHS	442	25,952	18,427	12,786	17	112	0.89	2.79	1.31	1.00
9	MQPSO	1106	29,973	30,352	11,430	18	188	0.91	2.66	1.31	0.87
10	NSGA-II	435	29,973	28,293	12,322	12	156	0.9	3.45	1.48	0.86
11	NSGA-II	580	21,931	18,400	14,838	11	155	0.9	3.22	1.56	1.00
12	MDE	414	29,973	10,242	14,259	17	112	0.87	3.18	1.56	1.00
13	MGP	448	25,952	22,849	12,684	12	114	0.89	3.65	1.59	0.97
14	MDE	385	33,994	14,707	14,104	14	150	0.91	3.95	1.68	0.86
15	MHS	411	36,187	18,320	12,847	14	177	0.83	3.75	1.70	1.00
16	nadir	1,405	36,187	33,259	14,838	11	112	0.83	7.00	2.64	1.00
17	ideal	385	21,931	10,242	9500	18	191	0.92	0.00	0.00	0.00

## Data Availability

Datasets such as Benchmark90, Benchmark306, Benchmark855 and Benchmark1020 are available on site https://www.researchgate.net/profile/Piotr-Dryja (owner Jerzy Balicki and Piotr Dryja) under CC BY license. Cite: Balicki, J.; Dryja P. Multi-objective tabu-based differential evolution for teleportation of smart virtual machines in private computing clouds. In Proceedings of the 2021 IEEE Congress on Evolutionary Computation (CEC), Kraków, Poland, 28 June–1 July 2021; pp. 1904–1911.
